# A three-dimensional printed model in preoperative consent for ventricular septal defect repair

**DOI:** 10.1186/s13019-021-01604-w

**Published:** 2021-08-11

**Authors:** Xicheng Deng, Siping He, Peng Huang, Jinwen Luo, Guangxian Yang, Bing Zhou, Yunbin Xiao

**Affiliations:** 1 Heart Center, Hunan Children’s Hospital, No. 86 Ziyuan Road, Changsha, 410007 China; 2 Department of Radiology, Hunan Children’s Hospital, Changsha, 410007 China; 3 Department of Radiology, The Second Xiangya Hospital, Central South University, Changsha, 410007 Hunan China

**Keywords:** 3D printing—heart defects, Congenital—surgical procedures, Operative—informed consent

## Abstract

**Background:**

The 3D printing technology in congenital cardiac surgery has been widely utilized to improve patients’ understanding of their disease. However, there has been no randomized controlled study on its usefulness in surgical consent for congenital heart disease repair.

**Methods:**

A randomized controlled study was performed during consent process in which guardians of candidates for ventricular septal defect repair were given detailed explanation of the anatomy, indication for surgery and potential complication and risks using 3D print ventricular septal defect model (n = 20) versus a conventional 2D diagram (n = 20). A questionnaire was finished by each guardian of the patients. Data collected from questionnaires as well as medical records were statistically analyzed.

**Results:**

Statistically significant improvements in ratings of understanding of ventricular septal defect anatomy (*p* = 0.02), and of the surgical procedure and potential complications (*p* = 0.02) were noted in the group that used the 3D model, though there was no difference in overall ratings of the consent process (*p* = 0.09). There was no difference in questionnaire score between subjects with different education levels. The clinical outcomes, as represented by the duration of intensive care unit stay, intubation duration was comparable between the two groups.

**Conclusions:**

The results indicated that it was an effective tool which may be used to consent for congenital heart surgery. Different education levels do not affect guardians’ understanding in consent. The impact of 3D printing used in this scenario on long term outcomes remains to be defined.

## Introduction

Physician–patient communication has long been considered one of the most important yet challenging components in the clinical setting [1]. Patient understanding of their diagnosis and treatment is important to their compliance and satisfaction with physicians [1, 2] and may have an impact on clinical outcomes. In view of this, visual aids are often used for communication with patients as they understand and retain information better with visual assistance [3]. Nonetheless, conventional graphical aids often require patients to imagine a three-dimensional structure in a two-dimensional form. This is particularly challenging when it comes to congenital heart defects (CHD) as the patient and family lack anatomic acknowledge and complex anatomy of a heart with congenital defects.

Three-dimensional (3D) printing has emerged as an invaluable tool to produce anatomic models with high fidelity. The spatial and anatomic complexity that can be replicated with this technology has enabled the reproduction of patient-specific replicas with high fidelity and has been widely used in medical education and training [4–8]. Studies [9–11] have shown the value of this technique in training and practices of healthcare providers of CHD, but its role in physician–patient communication remains to be defined.

Here we report the use of 3D printing technology to create an anatomic replica of a patient with a typical ventricular septal defect (VSD). This customized model had been designed to be sliced through the right ventricle to expose the defect during consent when surgeons employed the model, versus using traditional two-dimensional heart diagrams, as visual aids to evaluate their ability to improve patients’ subjective understanding of the anatomy, and proposed surgical procedures.

## Materials and methods

### Study design and selection of patients

This study was approved by the Institutional Review Board at our institution (Reference Number: HCHLL-2018-02). The study was prospective, two arms, single center, and randomized. The randomization of patients was performed using an equation established from rand() function in Microsoft Excel (Microsoft Corporation, Seattle, WA, USA). All subjects were enrolled and consented in the study by the same surgeon, followed by a questionnaire to fill out. Since our patients were all minors, their parents were approached for decision-making. From January 2018 to January 2019, guardians of 44 patient candidates for elective perimembranous VSD repair were approached, and 40 were included. Four were excluded for conversion to device closure (n = 1), refusal to participate outright (n = 2), or not being able to understand the study (n = 1). No included patient had previously undergone any interventional therapy or open-heart surgery. The control group had the information about their disease, the anatomy of the defect and surgical indications and potential complications explained by a surgeon using the current standard of clinical care, including 2D charts downloaded from the Internet and verbal explanation. The study group had the same information given to them using a 3D printed model of a heart with perimembranous type VSD to help explain the lesion’s anatomy, surgical procedure and potential complications. The same model was used throughout the study. Immediately after communication, a Likert-type questionnaire with 3 question items (the questionnaire was designed and approved by all authors and included understanding of VSD anatomy, of procedure and its complications, and overall understanding of the consent process focusing on verbal and visual aid-assisted communication) with a scale from 1 to 10 for each item as well as an additional item exclusively for experimental group regarding the usefulness of the 3D print from the guardians’ perspective was required to complete. The questionnaires also collected information of the guardian’s ages, genders and education level, which were self-reported by them. The whole process was conducted by the same surgeon throughout the study. Additionally, perioperative data of patients as well as other relevant information were collected from patient records.

### CT data processing and 3D printing

Pre-existing CT data obtained with a SOMATOM Force scanner (Siemens Healthineers, Munich, Germany) was used for 3D printed model production. The patient from whom the CT data was obtained was initially suspected to have coarctation of the aorta in addition to an explicit perimembranous VSD, though later CT scan dismissed this coarctation suspicion. CT slice thickness ranged from 0.5 to 1.0 mm and pixel spacing ranged from 0.5 to 1.0 mm. The CT data of a previous patient was selected and opened in Mimics software (Materialise, Leuven, Belgium). The segmentation was achieved using intensity value threshold followed with semi-automated and manual maneuver. The following blood volume subsets were segmented: left atrium with pulmonary veins, right atrium with vena cava, left ventricle, right ventricle, pulmonary arteries, aorta, and ascending aorta with coronary arteries. Additional segments included the VSD. All volumes outside of the volume of interest were masked. The result was exported from Mimics as a.stl file and imported for printing on an RS6000 printer (Alliance 3D Printing Technology, Shanghai, China) using photosensitive resin with a glossy finish.

### Statistical methods

PASS 15.0 (NCSS, LLC. Kaysville, Utah, USA) was used for sample size calculation before enrollment of the study. The following parameters were used: A mean questionnaire rating of 9.5 for 3D print and 8 for 2D charts arms, 1.0 for standard deviation for both arms, 0.90 for power, and 0.05 for Alpha. The sample size was calculated as 14 including drop-outs for each arm. Stata version 18.0 (Stata Corp, TX, USA) was used for statistical analyses. All values are reported as mean ± standard deviation or median with interquartile. Demographic and clinical characteristics between groups were compared using Student t test for normally distributed continuous variables. Wilcoxon rank-sum test was used for non-normally distributed variables and chi-square or Fisher's tests for categoric variables as appropriate. Results were considered statistically significant for *p* < 0.05.

### Ethical approval

This study was approved by the Institutional Review Board at our institution (Reference Number: HCHLL-2018-02). All research was performed in accordance with relevant guidelines/regulations. Written informed consent was waived by the ethics committee due to minimal risk and the need to prevent full disclosure that may have induced bias and participation was voluntary. The study did not involve any use of human tissue samples.

## Results

**Fig. 1:**
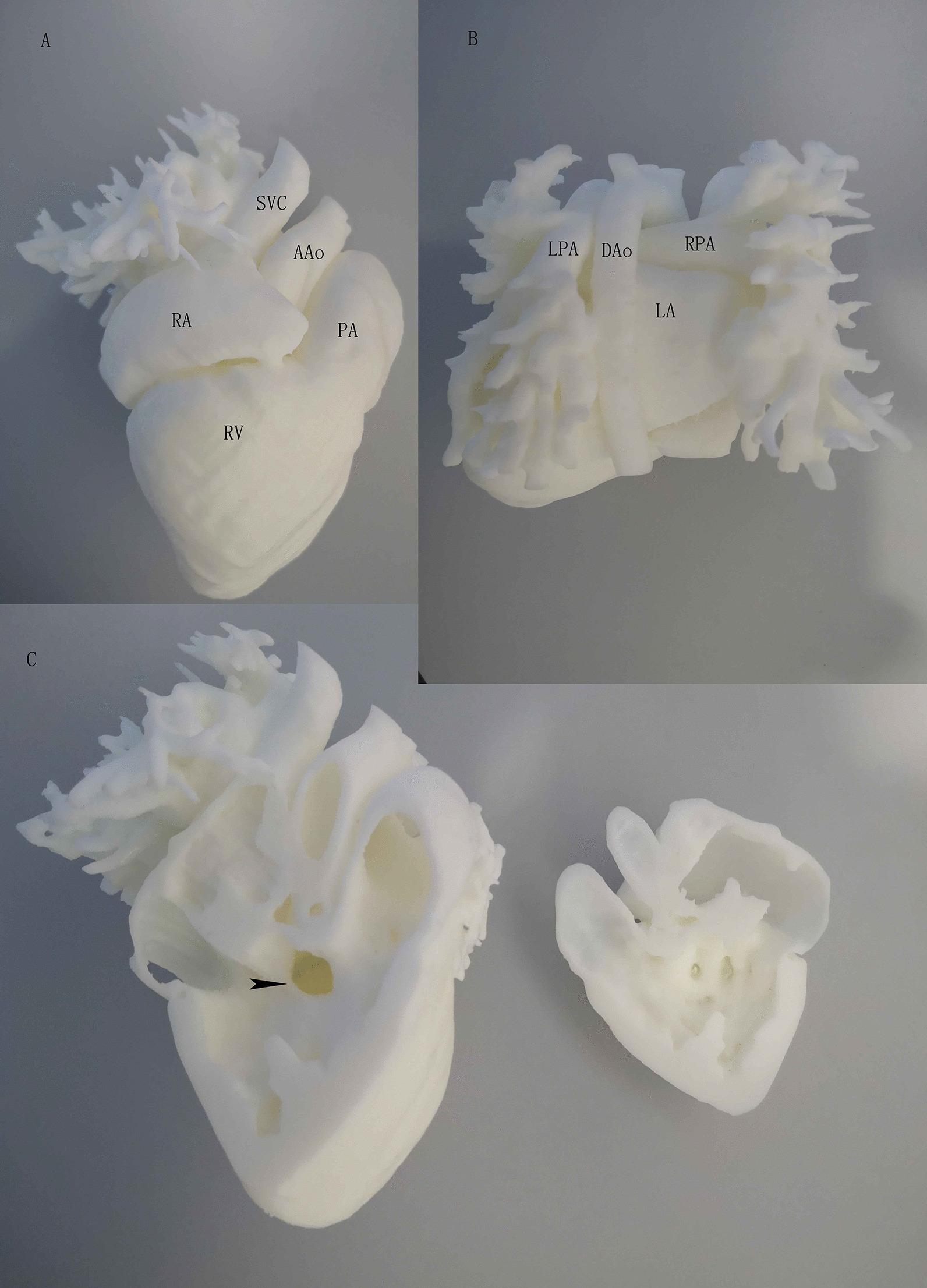
**A** Front view of the model. SVC: superior vena cava, PA: pulmonary artery, AAo: ascending aorta, RA: right atrium, RV: right ventricle. **B** Back view of the model. LPA: left palmary artery, DAo: descending aorta, RPA: right pulmonary artery, LA: left atrium. **C** Inside view of the model. Arrow head: ventricular septal defect

**Fig. 2:**
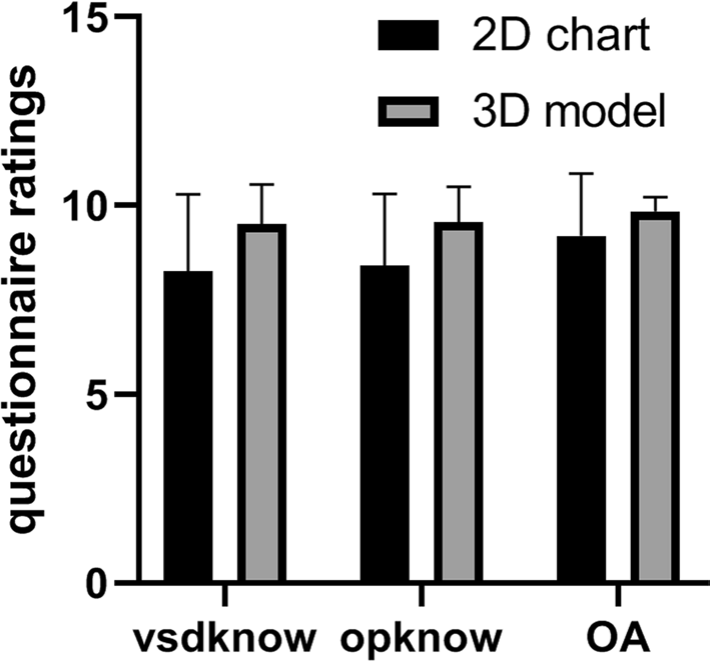
Comparison of questionnaire ratings between two groups. vsdknow: VSD knowledge, opknow: Operation knowledge, OA: Overall understanding

**Fig. 3:**
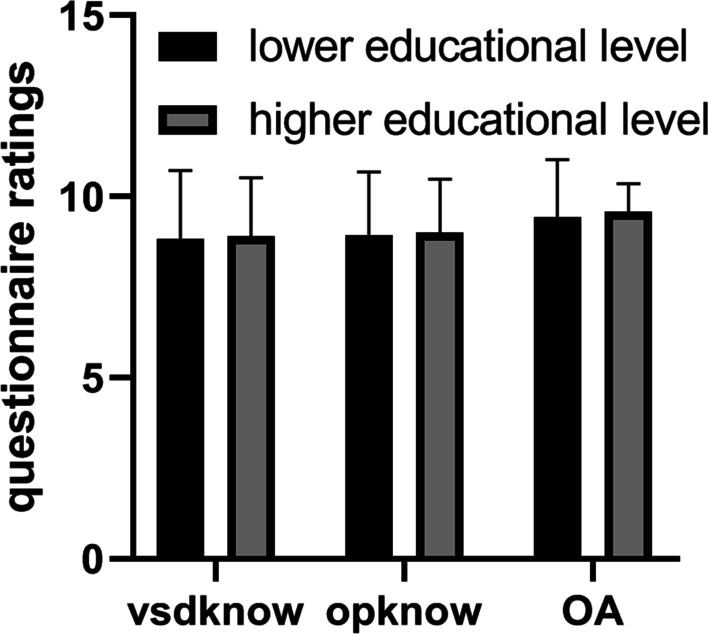
Comparison of questionnaire ratings between different education levels. vsdknow: VSD knowledge, opknow: Operation knowledge, OA: Overall understanding

A pure white color 3D printing heart with high fidelity was successfully produced. The model was designed to be sliced through the right ventricle in coronal plane with a 10 mm sized perimembranous VSD clearly visualized (Fig. 1). There was no difference between the two groups in parent demographics or education levels (Table 1). The data from questionnaire survey showed guardians’ understandings of both VSD and operation knowledge were significantly higher in 3D printed model arm, though there was no difference in overall ratings (Table 2 and Fig. 2). The usefulness of the model was also rated high at an average of 9.7 with only one participant giving a score of 6. We also investigated the effect of different education levels on questionnaire score and found no difference between low (including subjects with primary school and junior high school levels) and high (including subjects with senior high school and college/university levels) groups (Table 3 and Fig. 3).

**Table 1:** Guardians and questionnaire information

	3D model	2D chart	*p*
Duration of consent (min)	9.5 ± 3.6	8.6 ± 2.2	0.35
Age of guardians (years)	32.8 ± 6.8	32.0 ± 5.9	0.68
Gender of guardian (total (female))	20(2)	20(6)	0.11
Education of guardian level			0.26
1	2	0	
2	8	10	
3	4	7	
4	6	3	
VSD knowledge (1–10)	9.5 ± 1.1	8.3 ± 2	0.02
Operation knowledge (1–10)	9.6 ± 0.9	8.4 ± 1.9	0.02
Overall understanding (1–10)	9.9 ± 0.4	9.2 ± 1.6	0.09
Usefulness of 3D	9.7 ± 1.0		

Education of guardian level: 1: primary school, 2: junior high school, 3: senior high school, 4: college/university; VSD: ventricular septal defect

**Table 2:** Preoperative demographics and associated procedures of the patients

	3D model	2D chart	*p*
Gender, total(female)	20(10)	20(6)	0.20
Age(years)	0.3,0.3–1.1	0.6,0.4–2	0.31
Weight (kilograms)	5.4,4.3–8.3	6.7,4.7–9.5	0.28
Associated heart procedures			
PFO/ASD closure	17/3	16/4	1.00
PDA ligation	11	11	1.00
RVOT obstruction repair	0	1	1.00
Tricuspid valvuloplasty	5	10	0.19
Mitral valvuloplasty	0	1	1.00

*PFO* patent foramen ovale, *ASD* atrial septal defect, *PDA* patent ductus arteriosus, *RVOT* right ventricular outflow tract

**Table 3:** Questionnaire ratings by dichotomized educational level

	Lower educational level (n = 20)	Higher educational level (n = 20)	p
VSD knowledge	8.9 ± 1.9	8.9 ± 1.6	0.93
Operation knowledge	9.0 ± 1.7	9.0 ± 1.5	0.92
Overall understanding	9.5 ± 1.6	9.6 ± 0.8	0.70

*VSD* ventricular septal defect

## Discussion

Our results imply that providing guardians with a disease-specific 3D heart model for surgical consent is equivalent or superior to conventional 2D diagram in helping them understand the anatomy and treatment options of the disease. To the best of our knowledge, this is the first randomized controlled trial that has attempted to quantify the benefit of 3D print models in surgical consent. Three-dimensional printed heart replicas have been widely used in medical education [12, 13], training and planning [14] as well as clinical communication [15]. However, these studies were either descriptive or quantitative, but without control group studies, or involving patients with a heterogeneity of diseases. Though many studies of these demonstrated benefit from this technique, they seemed to be less powerful in terms of study design. Lim et al. [16] reported a randomized controlled trial comparing 3D prints versus cadaveric materials. This study was aimed at medical students and only involved normal cardiac anatomy. The merits of disease- or patient-specific 3D print have been confirmed in studies abovementioned. It results better understanding of 3D spatial and anatomic relationship of an organ or a structure, especially in a lesion with complex anatomy, compared to using conventional diagram or images [17]. Understanding of the surgical procedure is critical to quality of care and compliance.

The impact of education level of patients and guardians has been frequently studied previously. Thought some authors have found variances in perception or preference of communication [18, 19], most of them concluded the subjects’ understanding of information conveyed was not affected by different education levels [18–20]. In the current study, we also found there was no difference in ratings between guardians with lower and higher education levels, indicating 3D prints could be used in all guardians regardless of their education levels.

As for consent duration, one study [21] has shown 5 min on average longer communication time using 3D prints compared to our results showing no statistical difference in this aspect. This is possibly due to study variance, such as wording in explanation. In our study, same durations produced better understanding comparing to using 2D diagrams. This highlighted the efficiency of 3D prints in showing the spatial relationship in the heart with a defect. It is worth noting that there were shorter durations in our study than the previous one by Biglino and colleagues, being 9.5 ± 3.6 vs 21 ± 10 min and 8.6 ± 2.2 vs 16 ± 7 min for with 3D prints and 2D diagrams, respectively. The shorter times in our study may be explained by cultural and practice difference. A typical parent of a child with CHD is usually not well-education to understand the details of the operation and complications in China. The parents prefer to rely on and trust in surgeons by signing on consent form straightforward. Also, the indication, potential complication and expected outcomes usually are already explained in outpatient clinic before admission for surgery.

We only used pure white material to simply show the structure of the VSD. Studies [22, 23] have demonstrated color coding models may result in more intuitive representations. For example, blue and red rendering for veins and arteries, respectively, are often used in clinical medicine. We did not use multiple colors for economic issue. Further studies may incorporate multi-color labeling to confirm if this method produces better communication results.

The cost of patient-specific 3D print has dropped dramatically over time, but it is still significant. Since we in this study only included subjects with perimembranous VSD and only used one print, it was cost effective. When extended to complex CHD, there seems no one-size-fits-all solution and individualized 3D prints are inevitable. Though there have been multiple studies indicating the unique advantage of this technique in complex CHD [14, 24, 25], the cost remains a major obstacle for conventional application when this technique is brought to communication with patients or guardians with wider range of congenital cardiac defects.

### Limitations

As abovementioned, the study population is relatively small and only included perimembranous VSD subjects, limiting the generalizability of the results. Usually, CT or MRI scan is not used in diagnosing an isolated VSD for echocardiography is almost always enough and also to avoid unnecessary radiation or sedation, making data source for VSD 3D printing difficult to obtain. Since every lesion is unique, even for the same type of heart defect, the single 3D printed model obviously could not completely represent specific characters of each patient’s heart defect, such as variance in sizes, locations of the VSD. Moreover, the associated lesions, including patent foramen ovale, atrial septal defect, patent ductus arteriosus, right ventricular outflow tract stenosis, tricuspid and mitral regurgitation that need repair concomitantly could not always be well demonstrated on the model, especially the relationship between tricuspid valve and the defect due to poor image definition of valvular structures obtained from CT scan. We chose perimembranous VSD for study as it was the most common type of CHD. In view of the low incidence and wide variance of most of the other CHDs, it is difficult to use one replica for all patients of a specific lesion. The survey focused on young guardians’ response to the 3D models in a hospital setting, without any further follow-up, so the impact on post-discharge satisfaction of medical/surgical care or improved quality of life cannot be obtained. The consent process was also performed by the same surgeon who performed the operations, so there was inevitably a conflict of interest while filing the questionnaires for the family, though we consider it could have exerted the same effect in the two groups. In addition, to increase the response rate of the survey we did not objectively assess the impact of the models on learning and knowledge acquisition.

## Conclusions

We have demonstrated the usefulness of a 3D-printed model of perimembranous VSD for surgical consent. Different education levels do not affect guardians’ understanding in surgical consent. The impact of 3D print used in this scenario on clinical outcomes remains to be defined.

## Data Availability

Data can be provided upon request.

## Abbreviations

3D: Three -dimensional
2D: Two-dimensional
CHD: Congenital heart defects
VSD: Ventricular septal defect
CT: Computed tomography imaging
